# Model and Scenario Variations in Predicted Number of Generations of *Spodoptera litura* Fab. on Peanut during Future Climate Change Scenario

**DOI:** 10.1371/journal.pone.0116762

**Published:** 2015-02-11

**Authors:** Mathukumalli Srinivasa Rao, Pettem Swathi, Chitiprolu Anantha Rama Rao, K. V. Rao, B. M. K. Raju, Karlapudi Srinivas, Dammu Manimanjari, Mandapaka Maheswari

**Affiliations:** Central Research Institute for Dryland Agriculture (CRIDA), Hyderabad, India; USDA-Agricultural Research Service, UNITED STATES

## Abstract

The present study features the estimation of number of generations of tobacco caterpillar, *Spodoptera litura*. Fab. on peanut crop at six locations in India using MarkSim, which provides General Circulation Model (GCM) of future data on daily maximum (T.max), minimum (T.min) air temperatures from six models viz., BCCR-BCM2.0, CNRM-CM3, CSIRO-Mk3.5, ECHams5, INCM-CM3.0 and MIROC3.2 along with an ensemble of the six from three emission scenarios (A2, A1B and B1). This data was used to predict the future pest scenarios following the growing degree days approach in four different climate periods viz., Baseline-1975, Near future (NF) -2020, Distant future (DF)-2050 and Very Distant future (VDF)—2080. It is predicted that more generations would occur during the three future climate periods with significant variation among scenarios and models. Among the seven models, 1–2 additional generations were predicted during DF and VDF due to higher future temperatures in CNRM-CM3, ECHams5 & CSIRO-Mk3.5 models. The temperature projections of these models indicated that the generation time would decrease by 18–22% over baseline. Analysis of variance (ANOVA) was used to partition the variation in the predicted number of generations and generation time of *S. litura* on peanut during crop season. Geographical location explained 34% of the total variation in number of generations, followed by time period (26%), model (1.74%) and scenario (0.74%). The remaining 14% of the variation was explained by interactions. Increased number of generations and reduction of generation time across the six peanut growing locations of India suggest that the incidence of *S. litura* may increase due to projected increase in temperatures in future climate change periods.

## Introduction

The recent report of the Intergovernmental Panel on Climate Change (IPCC) reconfirmed that earth’s surface temperature has increased by 0.78°C (0.72°C to 0.85°C) between the average of the 1850–1990 period and the 2003–2012 period and the amount CO_2_ in the atmosphere has grown by about 40% over pre-industrial levels. The projected increase in temperature by 2100 was set at 1.8–4°C [1]. The predictions of temperatures vary with models and emission scenarios [2]. Agriculture is one of the sectors most vulnerable to anticipated climate change. The impact of climate change on agricultural production is unlikely to be evenly distributed across regions. However, in low-latitude regions, moderate temperature increases (1–2°C) are likely to have negative yield impacts for major cereals. Warming of more than 3°C would have negative impacts in all regions [3]. The changes in temperature will be uneven during a year and thus will have implications for crop growth and productivity. Increased temperatures can potentially affect insect survival, development and population size. Temperature can impact insect physiology and development directly or indirectly through the physiology or existence of hosts. Increased temperatures will accelerate the development of insects—possibly resulting in more generations. Low latitude and developing countries are likely to suffer more from agricultural effects of global warming, due to their disadvantaged geographical location, greater agricultural share in their economies, and limited ability to adapt to climate change. In contrast, crop production in high latitude regions will generally benefit from climate change. Recent global comprehensive estimate for over 100 countries, [4] predicted that global agricultural productivity would fall by 15.9% in the 2080s if global warming continues unabated, with developing countries experiencing a disproportionately larger decline of 19.7% [5]. Several global studies have indicated a loss of 10–40% in crop production in India by the end of the century [6]. Within India, dryland or rainfed agriculture is likely to suffer more because of the projected changes in rainfall and temperature.

Temperature is an important weather variable affecting dryland crops. The last three decades have seen a sharp rise mean annual temperature throughout India. Although most dryland crops tolerate high temperatures, rainfed crops grown during *rabi* are vulnerable to changes in minimum temperatures. Analysis of data for the period 1901–2005 by India Meteorological Department (IMD) suggests that annual mean temperature for the country as a whole has risen by 0.51°C over the period. Annual mean temperature has been consistently above normal (normal based on period 1961–1990) since 1993. This warming is primarily due to rise in maximum temperature across the country. However, since 1990, minimum temperature is steadily rising and rate of its rise is slightly more than that of maximum temperature [7]. Projections indicate the possibility of loss of 4–5 million tonnes in wheat production with every 1°C rise in temperature throughout the growing period with current land use [8].

Climate change resulting in increased temperature could impact insect pest populations in several complex ways. Temperature can impact insect physiology and development directly or indirectly through the physiology or existence of hosts. Increased temperatures will accelerate the development of many types of insects, possibly resulting in more generations (and crop damage) per year. The rate at which most pests develop is dependent on temperature and every species has a particular ‘threshold temperature’ above which development can occur, and below which development ceases. As temperature rises, some pest species may be able to complete more generations in a year. This effect may be most noticeable in insects with short life-cycles such as aphids and the diamond-back moth [9]. On the other hand, the temporary exposure of populations to extremely high temperatures may delay the development of surviving individuals and thus delay the subsequent generation.

Peanut (*Arachis hypogaea* L.), also known as groundnut, earthnut and ground bean, is the world’s fourth most important source of edible vegetable oil and the third most important source of vegetable protein. Peanut production is concentrated in Asia (50% of global area and 64% of global production) and Africa (46% of global area and 28% of global production), where the crop is grown mostly by smallholder farmers under rainfed conditions with limited inputs. China, India, Nigeria, USA and Myanmar are the major peanut growing countries. India is the second largest producer of peanut in the world with an average annual production of 5.51 million tons (http://faostat.fao.org) from an area of 5.47 million ha. Productivity of peanut in India is 1007 kg/ha as against 1522 kg/ha for the world and 3356 kg/ha for China. Elevated carbon dioxide (CO_2_) was reported to cause significant increase in total biomass at final harvest of peanut crop but decreased final seed yield in selected cultivars [10]. The crop is attacked by many species of insects which cause damage ranging from incidental feeding to near total plant destruction and yield loss [11]. Amongst them, the tobacco armyworm, *Spodoptera litura* (Fab.) is a major pest which can cause yield losses of 35–55%. Larvae feed gregariously on leaves causing severe defoliation, leaving only midrib veins.

The three emission scenarios viz., A2 (high emissions), A1 (medium emission-normally written A1B) and B1 (low emissions) are based on Green House Gases (GHG) emissions at three magnitudes [12]. Numerous weather generators such as Climate weather generator (Climgen), Weather Generator (WGEN), Long Aston Research Station (LARS-WG), MarkSim) are available for synthetic generation of data on weather variables such as temperature and precipitation. Utilization of weather generators has become essential for climate change studies as the GCM output is often given in terms of anomalies at monthly interval. In order to make use of the datasets for crop and pest modelling or prediction studies, it is essential that the information be temporally downscaled using weather generators. MarkSim GCM was developed to simulate weather from known sources of monthly climate data. It combines the spatial downscaling of weather data of selected GCMs to the point of interest and temporal downscaling to daily level. It provides six GCM Model results and an ensemble of the six models for three different emission scenarios.

The objective of the present study was to understand the impact of climate change on the number of generations of *S. litura* during future climate periods across major peanut growing locations in different states across India. In doing so, the study considered projections made using seven GCM model outputs and three different scenarios in order to get an insight in to the uncertainty or variation associated with climate change projections.

## Materials and Methods

### Future climate data

The future data on daily maximum (Tmax), minimum (Tmin) air temperatures, precipitation and radiation was obtained using MarkSim GSM (http://gismap.ciat.cgiar.org/MarkSimGCM). MarkSim typically calculates daily precipitation first and uses this information to guide the other weather variables such as daily solar radiation and maximum and minimum temperatures. MarkSim is a spatially explicit daily weather generator that was developed at International Centre for Tropical Agriculture (CIAT) and was released in 2004. It uses data from 10,000 stations in Latin America, 7000 in Africa and 4500 in Asia. MarkSim relies on climatic data surface interpolated from weather stations and generates long-term weather records on a grid basis of 18 km × 18 km. Each set of surfaces consists of monthly rainfall totals, monthly average temperature and monthly average diurnal temperature range. Thus the data from six models viz., BCCR-BCM2.0, CNRM-CM3, CSIRO-Mk3.5, ECHams5, INCM-CM3.0 and MIROC3.2 of three, A2, A1B and B1 was obtained. The main features of the six models along with the ensemble (AVG) can be found in another publication [13]. The details of these models and their abbreviations are given in the Table 1.

**Table 1:** Details and Abbreviations of Models.

**S. No.**	**Model**	**Code**
1	**BCCR-BCM 2.0**—Bjerknes Centre for Climate Research (University of Bergen, Norway	BCC
2	**CNRM-CM3**—Météo-France/Centre National de Recherches Météorologiques, France	CNR
3	**CSIRO-Mk3.5**—Commonwealth Scientific and Industrial Research Organisation (CSIRO) Atmospheric Research, Australia	CSI
4	**ECHam5**—Max Planck Institute for Meteorology, Germany	ECH
5	**INM-CM3.0**—Institute for Numerical Mathematics, Moscow, Russia	INM
6	**MIROC3.2**—Center for Climate System Research (CCSR), National Institute for Environmental Studies (NIES), and Frontier Research Center for Global Change (FRCGC), University of Tokyo, Japan.	MIR
7	**Ensemble (Average)**	AVG

MarkSim GCM provides daily data in a format compatible with Decision Support System for Agrotechnology Transfer (DSSAT) weather data files (http://dssat.net) and these files are downloaded to create new CLI files and WTG files for the simulated weather data under a range of GCMs and scenarios. In this study, we used the projections obtained at a resolution of 0.5 degrees from the original model resolution. The year of simulation was selected and simulated data was produced which would simulate the daily weather data representative of average of five years to either side of the year selected (10 year slice) [12]. The temperature data thus obtained were classified into four categories *viz*., Baseline-1975, Near future (NF)- 2020, Distant future (DF)- 2050 and Very Distant future (VDF)- 2080. The daily data during the crop duration of 133 days from 26^th^ to 44^th^ Standard Weeks were considered for predicting the number of generations and generation time of *S. litura*. MarkSim runs on interpolated climate grids to estimate the parameters of the model which produces simulated data files in .WTG format. The output of MarkSim does not represent series of years but replicate of the target year. The climate under particular situation of GCM/ scenario/year was obtained from 20 replicates. The .WTG file consists of data on four weather parameters viz., solar radiation, T.max, T.min and rainfall on daily basis in each replicate. These downloaded .WTG files were post processed to convert into .CSV files using Visual Basic based software ‘*INGEN*’ developed at CRIDA. After running the program, .WTG files are converted into 20 separate .CSV (Comma delimited) files along with two independent T.max and T.min files with compiled and arranged data of 20 replicates in primary phase. Later, these extracted T.max and T.min files were further processed by using INGEN for estimating the number of generations [14].

### Estimation of number of generations of *S. litura* on Peanut

Growing Degree Days (GDD) approach is used to predict the life cycle of insects in the form of number of generations during the season by measuring the growth in terms of temperature over time considering average daily temperatures which influence insect development. Expected number of generations were estimated using ‘*INGEN*’, wherein accumulated thermal degree days were calculated by horizontal cut-off (degree-day accumulations above the upper threshold do not count) method. The software provides data on GT (Generation Time) in days as given above, mean GDD (Mean Growing Degree Days—accumulated degree days to complete one generation after reaching cut-off GDD) and Total Degree Days (TDD—total summation of the degree days in a calendar year or crop season).

The standard GDD approach was followed to estimate the number of generations of *S. litura* occurring on peanut during a crop season. The maximum and minimum temperatures were transformed to heat units using the lower threshold temperature (t_0_) of 10°C for *S. litura* on peanut and the standard method for estimation of the degree days (thermal requirements) for each day was calculated by using the formula [15].

H=ΣD-D

Where:

H=Numberofheatunittoemergence

$$D-D=\left(T.max+T.min\right)/2–t_{0}$$

$$t_{0}=\;\mathrm{threshold}\;\mathrm{temperature}\;=\;10{}^{\circ}C$$

The minimum and maximum daily temperatures of different periods (BL, NF, DF and VDF) were used for estimating the accumulated thermal heat units. The number of degree days required for completing life cycle of *S. litura* (egg to adult) on peanut was taken as 522.7 DD [16], (www.nappfast.org/databases/) and GT generation time (average development time) needed for completion of one cycle was estimated. The number of generations of *S. litura* was calculated using cumulative degree days (Degree Day Units) for each generation of insect. Number of generations of *S. litura* and its generation time was predicted during entire crop season ranging from 26^th^–44^th^ standard week (swk) comprising of 133 days of crop duration across various locations [17]. This computation of degree days is different from that reported earlier in Ziter et al., [18] who accumulated the degree days for the entire calendar year. We considered the crop growth to occur only during the weeks comprising the current growing season in these areas of India. We did not consider that climate change might extend the growing season, as did Ziter et al., and so our results should be considered conservative estimates of the possible impacts of climate change on voltinism.

### Statistical analysis

The data on variation in number of generations of *S. litura* across six locations for the four periods *viz*., base, near, distant and very distant future periods were analyzed and the mean number of generations were compared using two-sample t-test assuming equal variances. The variation in the number of generations and generation time was decomposed by subjecting the data to ANOVA to understand the contribution of location, model, scenario and period to the total variation [18]. The sum of squares attributable to each source was divided by the total sum of squares explained by the model to obtain the individual contribution of each source. However, no statistical inference was drawn as the analysis was deterministic in nature. All statistical analyses were done using SPSS version 16.0.

## Results

### Variation in projected temperatures

The projected T.max and T.min of the six models along with their ensemble were obtained for six locations of the country and annual mean of temperature data of three emission scenarios was compared over baseline of 1975 and presented in Table 2. It is predicted that T.max would increase by 0.51 to 3.75°C and Tmin by 0.49 to 6.11°C during future climate change periods (NF, DF and VDF) of three emissions (A2, A1B and B1) at six locations of the country. The increase in temperature is higher during VDF period and expected to be in the range of 2.10°C (B1), 2.96°C (A1B) and 3.24°C (A2). Similar increase of temperature was reported [19] with projected annual increase of 2.06°C -3.63°C for the period 2071–2100 in other parts of Asia. It is well known that the prediction of future temperature varies with different GCM models across various parts of the world. The documented information on variation of temperature from three emission scenarios among seven models indicated more variation with CNR & ECH models which predicted an increase of 2–2.5°C in the average daily T.max and less with CSI (1.5–2°C) & MIR (1–1.5°C) models at other parts of Africa [20].

**Table 2:** Variation in annual mean of T.max and T.min of three emission scenarios across six peanut growing locations.

**Scenario/variable**	**Period**	**Bhubaneswar** (20^0^16’N, 85^0^50’E)	**Jalgaon** (21^0^5’N, 75^0^40’E)	**Junagadh** (21^0^31’N, 70^0^36’E)	**Raichur** (16^0^12’N, 77^0^36’E)	**Tirupathi** (13^0^ N, 79^0^E)	**Vridhacha-lam** (11^0^3’N, 79^0^26’E)
**T. Max**	1975	31.34	30.97	29.93	33.10	32.56	32.88
**A2 /T. Max**	2020	32.20	31.71	30.71	33.78	33.29	33.59
**A2 /T. Max**	2050	32.94	32.74	33.68	34.69	34.06	34.34
**A2 /T. Max**	2080	34.29	34.39	33.06	36.18	35.38	35.61
**A1B/ T. Max**	2020	32.36	31.84	30.79	33.90	33.35	33.62
**A1B/ T. Max**	2050	33.12	32.86	31.75	34.79	34.16	34.43
**A1B/ T. Max**	2080	34.08	34.11	32.88	35.84	35.14	35.35
**B1/ T. Max**	2020	31.85	31.71	29.11	33.79	33.76	33.42
**B1/ T. Max**	2050	32.85	32.51	31.33	34.49	33.88	34.12
**B1/ T. Max**	2080	33.39	33.23	31.97	35.06	34.41	34.62
**T. Min**	1975	22.77	17.61	21.14	22.17	22.34	23.84
**A2/ T. Min**	2020	23.26	18.64	22.00	23.13	23.26	24.70
**A2/ T. Min**	2050	24.14	19.91	22.88	28.28	24.22	25.61
**A2/ T. Min**	2080	25.53	21.83	24.50	25.97	25.74	27.03
**A1B / T. Min**	2020	23.44	18.83	22.09	23.31	23.39	24.81
**A1B / T. Min**	2050	24.32	20.08	23.10	24.41	24.37	25.74
**A1B / T. Min**	2080	25.30	21.44	24.24	25.57	25.44	26.74
**B1 / T. Min**	2020	23.63	18.68	19.74	22.98	23.29	25.20
**B1 / T. Min**	2050	23.97	19.54	22.67	23.95	23.94	25.32
**B1 / T. Min**	2080	24.50	20.30	23.31	24.61	24.54	25.90

Maximum Temperature (T. Max) Minimum Temperature (T. Min)

### Variation among models and scenarios

**Figure 1:**
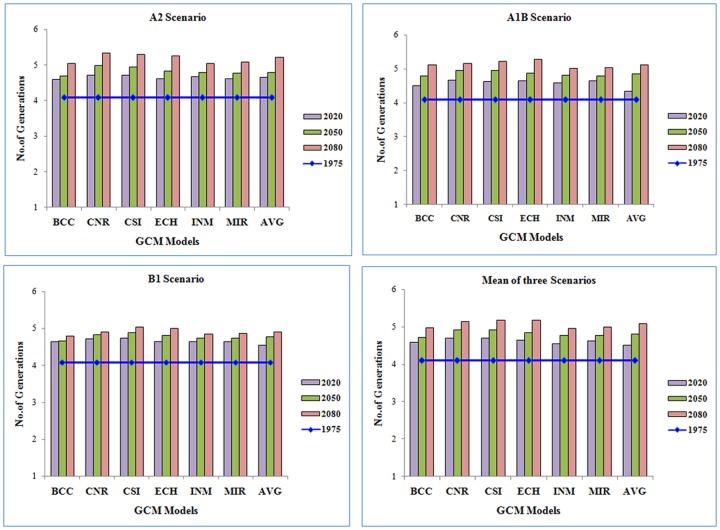
Inter Model and Scenario variation in number of generations of *S. litura* in future climate change scenarios.

The results on predicted number of generations during three future climate change periods viz., Near, Distant and Very Distant future using seven MarkSim models of three emission scenarios (A2, A1B & B1) are depicted in Fig. 1. In A2 scenario more generations with shortened generation time (4.71 with 26.81 days) are predicted by CNR model relative to the fewer generations and longer generation time predicted by the BCC model (4.59 with 28.02 days) in 2020.

**Figure 2:**
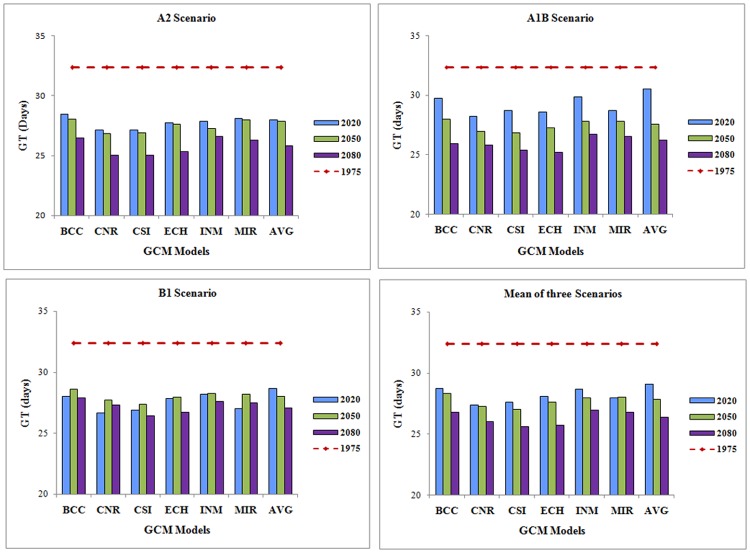
Inter model and Scenario variation in generation time of *S. litura* in future climate change scenarios.

The number of generations significantly increased during DF and VDF periods with CNR model temperature projections. More number of generations with reduced generation time (5.33 with 25.02 days) during VDF periods was predicted by majority of the models. In A1B scenario, more number of generations in crop season are predicted in case of ECH model (5.27 with 25.22 days) as against fewer number of generations with longer duration of generation in INM model (5.01 with 26.73 days) for 2080. In B1 scenario, more number of generations is expected when a temperature projection of CSI model is considered (5.03 with 26.42 days) over BCC model which predicted—fewer number of generations (4.79) with longer duration of generation (27.91 days) (Figs. 1 and 2).

### Percent change in generation time

**Figure 3:**
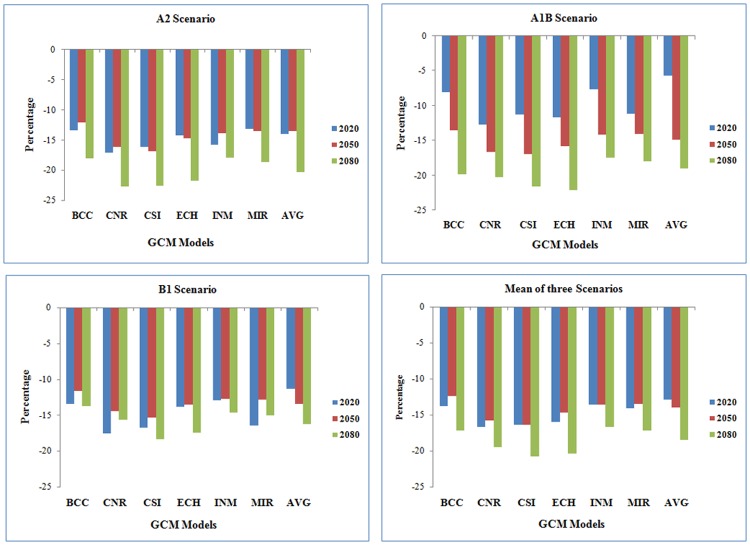
Per cent change in generation time of *S. litura* during future climate change across 7 models and 3 scenarios.

Our results indicate that the generation time of *S.litura* would decrease in future climate periods over baseline in each emission scenario. The percent reduction of generation time was calculated and is depicted in Fig. 3. The highest percent change in generation time is predicted during VDF (22.70) followed by DF (16.19) and NF (16.20) future climate change periods across three emission scenarios. Among seven models, the temperature projections of CNR and CSI models indicate that the generation time would shorten by 22.70 and 18.36% respectively over baseline. Generation time is expected to be shortened by 5.68 to 22.70% with all seven models at three emission scenarios. Similar trend was reflected even with mean of three emission scenarios (Fig. 3). It is predicted that more generations would occur during all three future climate change periods due to the reduction of generation time.

### Variation among scenarios across locations

In order to summarize the variation of number of generations and generation time of *S. litura* across six peanut growing locations of the country, the ensemble model data were used and the results are presented in Tables 3 and 4. In A2 scenario among six locations it is predicted that Vridhachalam location would experience higher number of generations of *S. litura* (5.61) with reduced generation time (23.55 days) in a crop season during VDF period followed by 5.29 generations of 25.04 days in DF and 5.16 generations of 25.18 days in NF period. In the other two emission scenarios similar trends were found at Vridhachalam location. Lower number of generations is predicted at Jalgaon location (4.77 generations with 29.65 days) during VDF period followed by 4.11 generations with 32.44 days and 4.01 generations with 29.58 days in DF and NF periods respectively. Similar trends were found from A1B and B1 scenarios as well (Tables 3 and 4).

**Table 3:** Inter Scenario variation in number of generations of *S. litura* in future climate change scenarios across peanut growing locations.

**Scenario/period**	**Bhubaneswar**	**Junagadh**	**Jalgaon**	**Raichur**	**Tirupathi**	**Vridhachalam**
**BL**	4.66±0.00	3.71±0.00	3.67±0.00	4.38±0.00	4.39±0.00	3.88±0.00
**A2/2020**	4.81±0.05*	4.01±0.05*	4.59±0.07*	4.51±0.05*	4.83±0.05*	5.16±0.05*
**A2/2050**	5.00±0.08*	4.78±0.13*	4.11±0.19*	4.75±0.10*	5.03±0.10*	5.29±0.09*
**A2/2080**	5.33±0.13*	5.14±0.98*	4.47±0.21*	5.12±0.14*	5.36±0.15*	5.61±0.14*
**A1B /2020**	4.81±0.06*	4.06±0.81*	4.85±0.24*	4.54±0.06*	4.54±0.18*	4.57±0.37*
**A1B /2050**	5.05±0.08*	4.82±0.11*	4.11±0.07*	4.78±0.07*	5.06±0.07*	5.32±0.07*
**A1B /2080**	5.28±0.13*	5.07±0.21*	4.50±0.11*	5.08±0.08*	5.30±0.12*	5.54±0.10*
**B1/2020**	4.82±0.50*	4.02±0.28*	4.60±0.03*	4.54±0.05*	4.87±0.05*	5.18±0.05*
**B1/2050**	4.96±0.08*	4.72±0.10*	4.03±0.06*	4.7±0.08*	4.99±0.08*	5.24±0.07*
**B1/2080**	5.09±0.10*	4.86±0.16*	4.18±0.10*	4.83±0.09*	5.11±0.06*	5.36±0.06*

* Indicate the statistical significance compared over baseline (BL) period at p<0.01.

**Table 4:** Inter Scenario variation in generation time of *S. litura* in future climate change scenarios across peanut growing locations.

**Scenario/period**	**Bhubaneswar**	**Junagadh**	**Jalgaon**	**Raichur**	**Tirupathi**	**Vridhachalam**
**BL**	28.35±0.00	35.87±0.00	35.85±0.00	30.45±0.00	29.66±0.00	34.02±0.00
**A2/2020**	24.9±2.89*	29.58±0.58*	28.92±1.20*	29.49±0.37*	27±0.36*	25.18±0.21*
**A2/2050**	26.47±0.40*	27.63±0.93*	32.43±0.87*	28.01±0.71*	26.35±0.53*	25.04±0.49*
**A2/2080**	24.88±0.69*	25.94±1.29*	29.65±0.47*	26.09±0.73*	24.66±0.79*	23.55±0.54*
**A1B /2020**	27.22±0.29*	32.05±4.81*	27.65±2.30*	29.28±0.45*	28.67±1.29*	29.02±2.64*
**A1B /2050**	26.30±0.42*	27.46±0.81*	32.16±0.63*	27.68±0.57*	26.22±0.39*	24.9±0.36*
**A1B /2080**	25.12±0.80*	26.21±1.52*	29.42±0.49*	26.15±0.41*	24.97±0.68*	23.78±0.51*
**B1/2020**	25.80±2.57*	29.80±2.02*	28.78±0.64*	29.28±0.34*	25.93±0.94*	25.04±0.25*
**B1/2050**	26.65±0.37*	28.21±0.74*	32.97±0.35*	28.37±0.52*	26.53±0.31*	25.30±0.38*
**B1/2080**	26.07±0.57*	27.28±1.22*	31.72±0.63*	27.61±0.57*	26.02±0.36*	24.7±0.34*

* Indicate the statistical significance compared over baseline (BL) period at p<0.01.

### Partitioned variation

**Figure 4:**
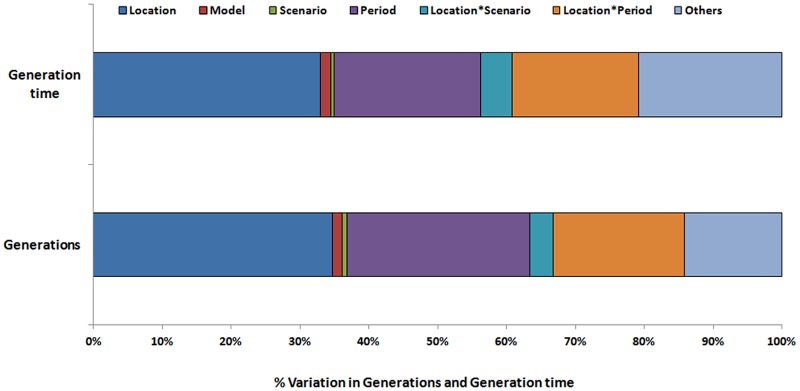
Relative proportion of variation in predicted number of generations and generation time of *S. litura* by model, location, period, scenario and others.

The partitioning of variation in number of generations are depicted in Fig. 4. The percentage of variation was in the descending order (from the source of uncertainty that explained the most variance in the results to the least) of geographical location > time period > location and time period interaction > model > scenario. These sources explained on average 34.7, 26.63, 19.07, 1.34 and 0.71% of the variation in the predicted number of generations of *S. litura* during peanut crop season respectively, with the remaining interaction terms explaining a combined 14.15%. Similar trend was noticed in generation time of *S. litura* also.

## Discussion

Temperature is the key factor which influences the growth and development of crop plants and herbivore insect pests and variations in temperature cause significant variation in these. Prediction of insect generations is possible by adopting growing degree days approach. Predictions of *S. litura* development on peanut were attempted under baseline and expected future climate change (NF, DF and VDF) periods. The accumulated thermal heat units expressed as degree days (DD), the number of generations and mean generation time of *S. litura* were estimated through selection of six representative peanut growing locations of India. The influence of temperature on development and survival of *S. litura* would affect the population dynamics of the pest and such influence can be estimated and quantified by calculating the number of generations [21]. The findings of present study indicate that 1–2 additional generations would occur during near and very distant future climate periods at six locations. Significant shortening of generation time resulted in occurrence of more generations at these locations. Number of generations varied among three emission scenarios and was more in A2 scenario (5.61) over A1B (5.54) and B1 (5.36) at Vridhachalam location indicating considerable inter scenario variation. Similar trend was observed at rest of the locations.

It is well known that accelerated development of insects is possible due to increased temperature which would result in more generations leading to greater crop losses during the season or year. Prediction of developmental time or mean generation time of insect pests in relation to temperature is an important tool for pest management [22]. Additional generations of multivoltine insect species (aphids and lepidopterans) are reported at higher temperatures [23]. Many of the lepidopteran species including *S. litura* would produce more generations under increased temperature conditions of near and distant future climate change period [21].

Production of more generations in a calendar year or during the crop season with higher temperatures is well known and is more evident with gradual warming [24]. Our studies on number of generations of *S. litura* on peanut in India with temperature data of 6 different models corroborate earlier research indicating that the insects respond to higher temperature with increased rates of development, more generations with less time between generations [25]. CNR and CSI models predicted more generations as they projected higher temperatures in future climate scenario. An upper threshold temperature of 37°C [16] was used in the study. The daily mean temperatures during peanut growing period of 133 days considered in this study ranging from 26^th^–44^th^ standard week (swk) rarely, if ever exceeds upper threshold temperature. However, if calendar year approach is followed there will be instances when daily mean temperatures exceed upper threshold and in such cases the effects of temperatures higher than upper threshold need to be considered while computing number of generations.

Among different sources of variation, geographical location explained higher percentage of variation (34.7%) followed by period (26.63%) which showed the significant effect of climate change on *S. litura*. In our study, considerable increase in minimum and maximum temperatures across six locations was observed during future periods over present. The interactions between location and period explained 19.07% of the variation. Models and scenarios explained very little of the variation which indicates that the changes in temperature and the resultant changes in number of generations are robust to the choice of model and scenario.

Ziter et al., [18] observed that for 13 insect pest species the choice of climate model explained an average of 42% of the total variation in voltinism, far more than did geographical location (33%), time period (17%) or scenario (1%). The remaining 7% of the variation was explained by various interactions, of which the location x GCM interaction was the strongest (5%). Further, regardless of these sources of uncertainty, a robust conclusion from above work was that all 13 pest species are likely to experience increases in the number of generations that they complete each year. In our findings geographical location explained higher per cent (34) of the total variation in number of generations, followed by time period (26%), model (1.74%) and scenario (0.74%). The remaining 14% of the variation was explained by interactions. We considered one insect pest of peanut whereas Ziter et al., [18] studied 13 insect pests. Further investigations are required after covering the all insect pests of peanut to determine the sensitivity of model, scenario, period and location. More variation is expected along the latitudinal gradient across locations indicating warmer temperature. The interaction between location and period indicates that the pest response would be partially period specific.

Significant variation in developmental time, number of generations and thermal requirements of *S. litura* on peanut with temperature data of six different models was predicted to occur during future climate change period. The present investigations were conducted considering the increase in temperatures alone although other factors such as precipitation, elevated CO_2_, crop phenology, pest phenology and multi-species interactions including pest—parasitoid associations under Climate Change scenario [18, 26] will also affect the growth and development of insect pests. Complete comprehension of confounding effects of climate change is possible only when this information on influence of other factors is available. The findings of studies using temperature alone are to be taken with these limitations in view.

## Supporting Information

S1 Data Final Data of PLOS ONE under Data Policy. (RAR)
